# Association between domestic violence and women's quality of life**^1^**

**DOI:** 10.1590/1518-8345.1535.2901

**Published:** 2017-06-05

**Authors:** Kerle Dayana Tavares de Lucena, Rodrigo Pinheiro de Toledo Vianna, João Agnaldo do Nascimento, Hemílio Fernandes Coelho Campos, Elaine Cristina Tôrres Oliveira

**Affiliations:** 2 PhD, Assistant Professor, Núcleo de Ciências Humanas, Sociais e Políticas Públicas, Universidade Estadual de Ciências da Saúde de Alagoas, Maceió, AL, Brazil.; 3 PhD, Adjunct Professor, Departamento de Nutrição, Universidade Federal da Paraíba, João Pessoa, PB, Brazil.; 4 PhD, Adjunct Professor, Departamento de Estatística, Universidade Federal da Paraíba, João Pessoa, PB, Brazil.; 5 MSc, Assistant Professor, Núcleo de Ciências Humanas, Sociais e Políticas Públicas, Universidade Estadual de Ciências da Saúde de Alagoas, Maceió, AL, Brazil.

**Keywords:** Gender Identity, Violence Against Women, Quality of Life

## Abstract

**Objective:**

to analyze the association between domestic violence against women and quality of life.

**Method:**

a cross-sectional population-based household survey conducted with women 18 years and older, using a stratified sample by neighborhoods. For analysis, prevalence of domestic violence and quality of life index was verified and logistic regression was used to determine associations, with a significance level of 5%.

**Results:**

424 women who had a prevalence of domestic violence of 54.4% and a quality of life index of 61.59 participated in this study. It was verified, through logistic regression, that domestic violence is associated with women's quality of life (p=0,017). The observed variables that influence the occurrence of domestic violence were in the social relations domain (p=0,000), provision of medical treatment for women (p=0,019) and safety (p=0,006).

**Conclusion:**

the study confirmed the evidence of an association between domestic violence against women and quality of life, a situation that reaffirms the importance of constructing public policies focused on gender emancipation.

## Introduction

Domestic violence, understood as synonymous with violence against women, is characterized as a phenomenon of multiple determinations and it is defined as any act based on gender relations that results in women's physical and psychological damages or suffering^1^. It refers to the hierarchy of power, desires of domination and annihilation of the other and that can be used consciously sometimes in marital relations as a mechanism for subordination of women to their partners^2^.

This type of violence against women has a social distribution around the world, being indicated as a kind of universal violence, practiced preeminently by partners or people very close to women^2^. According to data from the World Health Organization, in 2013, violence against women perpetrated by intimate partners showed a prevalence of 30%^3^.

In Brazil, according to data from the Map of Violence 2015, the expression of domestic violence against women (VDCM - in Portuguese) between 1980 and 2013, presented an upward trend, both in number and in rates. It was observed that a total of 106,093 women died victims of homicide in this period. The situation is worrying as the number of victims increased from 1,353 women in 1980 to 4,762 in 2013, an increase of 252%. This means that the rate of female victims of violence rose from 2.3 per 100,000 in 1980 to 4.8 per 100,000 in 2013 (an increase of 111.1%)^4^.

This high prevalence of violence against women -a phenomenon recognized as a public health problem^5^, has aroused the interest of society because of the serious consequences it can have on women's lives and the direct impact on their health even when it does not cause death due to injuries and physical or emotional trauma.

It is well known that violence perpetrated against women accompanies humanity in its historical course and presents different contents and forms in different societies. This type of violence often takes place in the private domain, and the home that may otherwise be the reference of refuge and protection, is in these cases a privileged place for the practice and concealment of violence. The risk of a woman suffering some form of aggression within the domestic environment is greater than in other vulnerable groups^6^.

In spite of its high prevalence and risk of violence, health care offered to women in situations of gender violence is still unsatisfactory^7^. This situation stems from the invisibility of the phenomenon in some sectors, such as emergency hospitals, most of which do not yet have ways to identify the problem, evidencing the predominance of the biologic model of health care, whose object of intervention is the physical damage^8^.

Data on VDCM in the state of Paraiba and in the municipality of Joao Pessoa are not organized in a database that facilitates the work of researchers and guides the authorities to make decisions in order to prevent or minimize the effects of violence against women. Additionally the data in the Public Security Secretariat of the state of Paraiba do not coincide with the existing data in the Department of Health of the Municipality of Joao Pessoa^9^.

Thus, to identify factors related to violence against women, as well as to deepen the discussions about the impact of this phenomenon becomes essential to guide the planning of governmental actions and to generate indicators of comparison among the different communities, regions and countries. For this purpose there is a need of research studies of these events in the communities focusing above all their influence on quality of life (QoL).

The concept of QoL defined by the World Health Organization (WHO) as a right of citizenship, referring to the sum of the economic, environmental, scientific-cultural and political conditions collectively constructed and put at the disposition of the individuals so that they can realize their potentialities^10^.

As the concept of QoL is dynamic, broad and subjective and resulting from the sum of factors arising from the interaction between society and the environment, it is important to understand how domestic violence affects the QoL of victimized women.

 Thus, the lack of knowledge about the association between domestic violence against women and quality of life is a research problem.

In view of the above, the present study aimed to analyze the association between domestic violence against women and quality of life given the existing opportunity to design and experiment with decision models that can give visibility to the VDCM phenomenon. It try as well to assist managers in the decision-making process in the construction and implementation of new effective public policies in addressing the problem, promoting a better quality of life for them through the evidence of the influence of explanatory variables on the occurrence of the phenomenon, considering for this the adjustment of a logistic regression model.

## Method

This is a cross-sectional population-based household survey conducted in the municipality of Joao Pessoa-PB from August 2013 to December 2015. The population was composed of women over 18 years of age living in this municipality. The inclusion criteria for this study were households in the city of Joao Pessoa, with female, 18 years old + dwellers. Exclusion criteria were: households with deaf-mute women; households without family conformation and households where the woman did not have the cognitive ability to answer the questionnaire.

The sampling plan used was stratified sampling by neighborhoods of the city of Joao Pessoa-PB. The target population was defined as all households in the city. The Municipality of Joao Pessoa, using 2014 as a reference, provided the list of these households. The selection of the sample was performed according to an optimal allocation method to the number of households per neighborhood, considering the fixed selection cost for all elements of the target population. The team of researchers checked blocks in each neighborhood and all households where women lived were included in the sample.

For the calculation of the sample, a total number of households belonging to the target population (143.479), total number of districts of Joao Pessoa (64), number of households belonging to the neighborhood (*h= 1,...64*), percentage of neighborhood households, number of households of the target population of the sample, belonging to the neighborhood, variable of interest (outcome) For the woman interviewed in the i-th individual selected in the neighborhood (1= wife of the i-th district domicile suffered violence; 0= woman of the i-th district domicile did not suffer violence), population reference value considering sample size^8^, assuming an error of 4.5% and a confidence limit of 95%. Possible losses were also considered during the execution of the study (10%). Thus, the sample size was 403 domiciles, from them a woman was interviewed according to the criteria of inclusion and exclusion of the research. However, due to the fact that several researchers were in the field, at the end of the collection 427 interviews were obtained.

Interviewers who underwent previous training performed data collection. They met the following conditions: being university students in the health area and having at least eight hours a week available for collection. Visits were carried out in the selected households and the collection of information obtained after clarifying the objectives of the research and signing the Informed Consent Form (ICF).

For the data collection, the following validated instruments were used: *WHO VAW STUDY* ^11^ to estimate gender-based violence against women and *WHOQOL BREF* for quality of life assessment^12^, as well as a semi-structured script containing sociodemographic and economic information (Age, marital status, level of education, race / color (self-reported), presence of children, employment status.

The instrument *WHOQOL-BREF* is composed of 26 questions, two of them about self-assessment of QoL and 24 issues representing each facet of the *WHOQOL-100*. For the composition of the questions of the *WHOQOL-BREF* the question of each facet that had the highest correlation with the average score of all facets was selected.

Thus, the *WHOQOL-BREF* is composed of four domains: Domain 1 (Physical), Domain 2 (Psychological), Domain 3 (Social relationships) and Domain 4 (Environment).

### Statistical analysis

Statistical analysis and information were obtained with the aid of statistical software R (version 2.14.1). A descriptive analysis was performed, based on absolute and relative frequencies for sociodemographic and economic variables; VDCM and QoL, in addition to the mean for continuous variables. The prevalence of VCMD was estimated and then the statistical method of logistic regression was used to identify the associated variables, since the response variable (outcome) presented previously is dichotomous, that is:



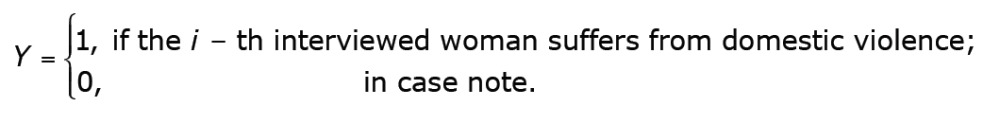



At this stage a significance level of 10% was considered. For the adjustment of the final model a significance level of 5% was considered.

The general test was applied (*Omnibus tests*) showing that the explanatory variables used in the model can calculate the probability of occurrence of VDCM (*p* value< 0,0001). The Nagelkerke R^2^ coefficient was then calculated, which value was 19%, showing that, with these explanatory variables, 19% of the total variability (scale de 0 a 100%) is reached. This value suggests that future research includes other variables in the model, since some variables were excluded by the method *Stepwise*.

Finally, the Hosmer-Lemeshow test was used. (valor *p* = 0,095), which in this case, accepted the null hypothesis that this model is compatible with the data, i.e. there is enough evidence to say that the predictions obtained with it are statistically equal to those observed in reality^13^.

### Ethical aspects

All female participants signed the ICF after receiving verbal and written explanations regarding the study. The research was approved by the Ethics and Research Committee of Lauro Wanderley University Hospital and obtained its approval under CAAE n° 20418813.0.0000.5183 in August 2013.

## Results

The sample consisted initially of 427 women, however, three questionnaires were excluded because they did not meet the inclusion criteria, thus the analysis comprised 424 women with an average age of 35 years. It was observed, in relation to sociodemographic and economic variables, that 56.0% of the women lived with a partner (36.0% married, 20.0% consensual union), 34,0% completed elementary school, 48,0% of the women declared themselves as white, 80,0% reported having children and 70,0% were employed. An overall prevalence of 54,4% of VCMD was estimated and a quality of life index (IQoL) of 61,59 (scale of 0 to 100). 

In Table 1 it is possible to observe, after adjustment of the logistic regression model and application of the method *Stepwise*, That the variables considered significant to explain the probability of occurrence of domestic violence were evaluation of the satisfaction of the QoL, domain 3 (social relationships), need for medical treatment and sense of safety.

**Table 1:** Results of the final adjustment of the logistic regression considering the scores of the 4 domains of quality of life. Joao Pessoa, PB, Brazil, 2015

Variables	Estimates of coefficients	Default error	p-value	OR*	CI**^†^** 95%
Satisfaction assessment of Qol**^‡^**	-0,058	0,024	0,017	0,943	0,899-0,990
Domain 3 (Social relationships)	-0,041	0,009	0,000	0,960	0,943-0,977
Need for medical treatment	0,202	0,086	0,019	1,224	1,033-1,450
Feeling of safety	-0,286	0,104	0,006	0,751	0,612-0,922
Intercept	3,334	0,702	0,000	-	-

*OR: Odds Ratio; †CI: Confidence interval; ‡QoL: Quality of life.

It is observed in Table 1 that the higher the evaluation of QoL satisfaction among women, their insertion in social relationships and their sense of security, the lower the probability of domestic violence against women, and the evaluation of QoL is a protection factor when considering the outcome. In other words, it was verified that women with an increased QoL score presented a 6.0% lower chance of suffering domestic violence. Women with increased insertion in their social relationships presented a 4.2% lower chance of suffering domestic violence and women with an increased sense of security presented a 33.2% lower chance of suffering domestic violence

It was also observed that as women's need of health care increases, the greater the probability of occurrence of violence, and the need for treatment is a risk factor related to the outcome. In terms of the odds ratio, it is verified that women increases their chances of suffering violence by 22.4% when the evaluation of their health satisfaction decreases.

After using the Logistic Regression Model, a comparison was made between groups of women who suffered some type of violence and a group of women who did not suffer any type of violence, considering the scores of the four quality of life domains, as well as The IQoL (function of the scores of the 4 domains).

To verify the normal distribution of data on domestic violence against women, we applied tests Lilliefors and Shapiro-Wilks^13^, and when analyzing the general quality of life index (IQoL), it was verified for both tests that there is evidence to affirm that the IQoL distribution is normal, but the distribution of the scores of the other domains is not normal, as presented in Table 2.

**Table 2:** Results of the normality tests of quality of life scores of women who underwent VDCM* with those who have not suffered. Joao Pessoa, PB, Brazil, 2015

Variable	Lilliefors (teste KS)			Shapiro-Wilk	
	Test statistic value	p-value		Test statistic value	p-value
Domain Score 1 (Physical)	0,088	0,000**^†^**		0,974	0,000**^†^**
Domain Score 2 (Psychological)	0,070	0,000**^†^**		0,987	0,000**^†^**
Domain Score 3 (Social relationships)	0,145	0,000**^†^**		0,930	0,000**^†^**
Domain Score 4 (Environment)	0,087	0,000**^†^**		0,982	0,000**^†^**
IQoL**^‡^**	0,031	0,390		0,997	0,431

*VDCM- Domestic violence against women; †Association statistically significant (p < 0,001); ‡IQoL: General index of quality of life.

Given that the normality tests used provided evidence that the IQoL variable is a normal distribution, the next step was to use a t test to compare IQoL averages for groups of women who have and have not experienced violence, respectively.

In relation to the other domains, since the normality tests used provided evidence that the distribution of the scores of these domains does not have normal distribution, the nonparametric test of Wilcoxon (Test W) to evaluate if the data scores from the two groups are not homogeneous. The test results are presented in Table 3.

**Table 3:** Results of the hypothesis test to compare the groups of women who suffered VDCM* with those who have not suffered VDCM. Joao Pessoa, PB, Brazil, 2015

Variable	Averages		p-value
	Group 1 (women who suffered violence)	Group 2 (women who did not experience domestic violence)	
Domain Score 1 (Physical)	65,33	70,59	0,0002**^†^** (Test W)
Domain Score 2 (Psychological)	60,13	67,31	0,0000**^†^** (Test W)
Domain Score 3 (Social Relationships)	64,71	75,37	0,0000**^†^** (Test W)
Domain Score 4 (Environment)	48,23	53,90	0,0002**^†^** (Test W)
IQoL**^‡^**	59,62	66,80	0,0000**^†^** (Test t)

*VDCM-Domestic violence against women;†A statistically significant association (p < 0,001); ‡IQoL: General index of quality of life.

The results of the group comparison tests show that, for all the individual domains scores as well as the overall quality score, there is statistical significance showing a difference between the groups of women who suffered and who did not suffer domestic violence.

When IQoL was observed, it was possible to verify that the mean of this score was higher in the group of women who reported not having suffered any type of violence. For the other domain scores, the interpretation is analogous, but since it was considered a non-parametric test, it can be said that the two groups are heterogeneous in relation to each of the scores.

## Discussion

It can be observed in this study that domestic violence is a reality, and highly prevalent in the daily life of women affecting their health and quality of life. This result corroborates data released by the World Health Organization (WHO)^2^ which evidenced the absence of a country or city immune to violence. In a pioneering research that gathered data from 35 studies in 24 countries^14^, the high incidence of violence of men against women was shown to be the most endemic form the sexual and physical violence of intimate partners against their women, which reiterates the phenomenon as a serious public health problem.

It should be pointed out that the higher rates of VDCM identification might relate to their greater transparency and less acceptance, due to the activist movements to confront violence. However, despite this progress, women still live in their daily social relationships, veiled social and symbolic situations affecting their psychological and physical health as well as their quality of life. 

When VDCM and QoL were associated, it was found through the evidence provided by the logistic regression model, that the domain "social relations" was statistically significant implying that women with social support network suffer less domestic violence when compared to those that do not present strong insertion in their social relations. The social life on the reciprocity and solidarity factors sustains the basis of social relations and its absence affects the quality of the relationship, increasing intolerance expressed in conflict resolution^15^.

It was observed in this study that the search for medical treatment also obtained statistical relevance with regard to the VDCM and its influence on the QoL of the women. The VDCM has a specific approach since an aggressor intimately related with the victim practices it. The use of physical force and/or psychological containment imposed on a woman against her interests, wants and desires, results in damage to physical and mental health by the violation of human dignity in its integrity^8^.

A study on quality of life and the presence of diseases such as depression in women suffering from violence revealed that most of the victims of aggression (72.0%) developed a significant degree of clinical depression. The majority of women experiencing violence (78.0%) also present symptoms such as anxiety and insomnia and those who suffered aggression (24.0%) began to use anxiolytic medication after the onset of episodes^16^.

An important finding observed in the same research is the fact that 39.0% of women who suffer violence have already considered suicide, which shows that VDCM causes effects such as poor quality of life and damages to their mental health^16^.

In this study it was observed that the variable "safety and protection" is involved with VDCM and influences QoL. It is known that despite the existence of the "Maria da Penha" Brazilian Law, many women do not feel safe and protected in order to denounce the aggressor. This insecurity relates to the difficulty of reacting and verbalizing violence, fear for their own safety and for their children, lack of control over their lives, the hope that the aggressor will change or the willingness to protect the partner for economic or affective reasons^8^.

A study that analyzed the trajectory of coping with the violence carried out by some women showed that denouncing or deciding to end their silence in the face of the situation of violence presents both facilitating and troublesome aspects. Among the facilitating aspects are personal attitudes, such as feelings of exhaustion and revolt, and awareness of potential life risks. Among the troublesome factors, fear, guilt and shame, as well as family, material and institutional obstacles^17^.

It is important to emphasize that institutional obstacles refer to the inefficiency of the system in guaranteeing the protection of women against their aggressor, the lack of preparation and bureaucracy of the legal system, the lack of preparation of health professionals to deal with situations of violence and lack of monitoring and accountability^18^.

The instrumental knowledge that guides the professional practices is geared towards treating above all, the physical damages, thus excluding feelings and subjectivity. This inadequacy of instrumental knowledge constitutes an obstacle to the development of a health work process that changes this reality.

Research studies in health services^18^ about VDCM, identified that the expectation of women when seeking the health service is to be heard and welcomed, a situation that is hardly effective. The justifications are focused on barriers created in the service, either for lack of time and resources, fear of offending women, lack of qualification or even frustration for not having the response of many users to the advice they receive.

The VDCM phenomenon is one of the major problems of public health, considering that they are intimately related to the morbidity in the female population. A study conducted in Colombia found that intimate partner violence resulted in symptoms such as chronic pain and depressive events^19^. In Brazil, a study carried out in Espirito Santo, found that more than half of the women who suffered violence began to perceive their state of health as rather bad or poor only after a traumatic event. They reported pain, inadequate sleep and fatigue all the time, easily scared, easily nervous, tense or worried and crying more than usual^20^.

As for the state of Paraiba, in the year 2013, it occupied the eighth position among the Brazilian states with the highest incidence of deaths of women by violence. It was observed that 29.0% of these deaths occurred at home^21^. Data from the Nucleus of Criminal and Statistical Analysis (NACE) of the Secretariat of Social Security and Defense (SEDS) of the state of Paraiba indicated that in that same year there were 118 cases of female homicides^22^. According to the March 8th Women's Center, of these 118 homicides, 35 were motivated by domestic violence. 

It can be seen from the statistics found in the present study that there is evidence that the VDCM directly and negatively affects the quality of life in several aspects, since it interferes in the physical and psychological health of women, in society and their social relations and also for the health system.

Considering the complexity of the VDCM, which can be gradually changed over the generations, it is emphasized that any change in behavior involves reflection, questioning in various social instances (family, school, work, church, among others). These changes in the way of thinking and acting can lead to the formation of a new social order.

But we still need to overcome challenges involving VDCM. Difficulties in overcoming such challenges include obstacles to its diagnosis, such as cultural factors, the lack of orientation of users and health professionals, and the subjects involved who are afraid to deal with the unfolding of the phenomenon.

Unfortunately, only gender equality is not enough to match women socially, but it can be a starting point for their emancipation. These women make up the majority of the demand for public health services and it is in these places that they seek to be welcomed and cared for their health needs.

The State, as the defining body of public policies, must articulate professional practices in health, social superstructure and quality of life. This approach requires the consideration of interdisciplinarity and presupposes the use of mechanisms and instruments necessary for its attainment, among which are the Model of Decision Making. 

The recurrence of innovative technologies and solutions supports the decision-making process in public health, in the planning and development of policies that seek effective responses, aimed at prevention and / or control of health damage, to improve the quality of life of people and the social conditions of cities. This recurrence has intensified, especially after the expansion of the concept of health and the legal and political situation of considering Health as being among citizens's right and a duty of the Brazilian state.

Among the limitations observed in this study, it should be considered that this issue constitutes a challenge to be faced due to factors that may contribute to its underestimation such as: complexity of the subject, emotional condition of women in situations of violence, researcher's ability and instrument used. However, the VDCM-QoL association stands out as a relevant and usable finding for care in the prevention of the overall health of these women.

## Conclusion

The study showed evidence of an association between domestic violence against women and quality of life when considering the following explanatory variables: social relations domain and security and medical treatment offerings, which reaffirm the importance of building public policies focused on gender emancipation.

With regard to quality of life, it was evidenced from the hypothesis tests that the quality of life index of women who suffered domestic violence was lower (59.62) than the index of women who reported not suffering domestic violence (66.80).

This work presents how powerful it can be to use new strategies and approaches to discuss the problem of domestic violence against women, associating it with quality of life, in order to stimulate debates, to awaken new developments and future possibilities.
